# Genome-Wide Association Studies of Conotruncal Heart Defects with Normally Related Great Vessels in the United States

**DOI:** 10.3390/genes12071030

**Published:** 2021-07-01

**Authors:** Omobola O. Oluwafemi, Fadi I. Musfee, Laura E. Mitchell, Elizabeth Goldmuntz, Hongbo M. Xie, Hakon Hakonarson, Bernice E. Morrow, Tingwei Guo, Deanne M. Taylor, Donna M. McDonald-McGinn, Beverly S. Emanuel, A. J. Agopian

**Affiliations:** 1 Department of Epidemiology, Human Genetics and Environmental Sciences, UTHealth School of Public Health, Houston, TX 77030, USA; omobola.o.oluwafemi@uth.tmc.edu (O.O.O.); Fadi.I.Musfee@uth.tmc.edu (F.I.M.); Laura.E.Mitchell@uth.tmc.edu (L.E.M.); 2 Division of Cardiology, Children’s Hospital of Philadelphia, Philadelphia, PA 19104, USA; goldmuntz@chop.edu; 3 Department of Pediatrics, University of Pennsylvania Perelman School of Medicine, Philadelphia, PA 19104, USA; hakonarson@email.chop.edu (H.H.); taylordm@chop.edu (D.M.T.); emanuel@chop.edu (B.S.E.); 4 Department of Biomedical and Health Informatics, Children’s Hospital of Philadelphia, Philadelphia, PA 19104, USA; xiem1@chop.edu; 5 Center for Applied Genomics, Children’s Hospital of Philadelphia, Philadelphia, PA 19104, USA; 6 Department of Genetics, Albert Einstein College of Medicine, Bronx, NY 10461, USA; bernice.morrow@einsteinmed.org (B.E.M.); tingweiguo@gmail.com (T.G.); MCGINN@chop.edu (D.M.M.-M.); 7 Division of Human Genetics, Children’s Hospital of Philadelphia, Philadelphia, PA 19104, USA

**Keywords:** heart defects, congenital, genome-wide association study

## Abstract

Conotruncal defects with normally related great vessels (CTD-NRGVs) occur in both patients with and without 22q11.2 deletion syndrome (22q11.2DS), but it is unclear to what extent the genetically complex etiologies of these heart defects may overlap across these two groups, potentially involving variation within and/or outside of the 22q11.2 region. To explore this potential overlap, we conducted genome-wide SNP-level, gene-level, and gene set analyses using common variants, separately in each of five cohorts, including two with 22q11.2DS (*N* = 1472 total cases) and three without 22q11.2DS (*N* = 935 total cases). Results from the SNP-level analyses were combined in meta-analyses, and summary statistics from these analyses were also used in gene and gene set analyses. Across all these analyses, no association was significant after correction for multiple comparisons. However, several SNPs, genes, and gene sets with suggestive evidence of association were identified. For common inherited variants, we did not identify strong evidence for shared genomic mechanisms for CTD-NRGVs across individuals with and without 22q11.2 deletions. Nevertheless, several of our top gene-level and gene set results have been linked to cardiogenesis and may represent candidates for future work.

## 1. Introduction

Congenital heart defects comprise some of the most common, serious, and clinically important groups/types of birth defects [1,2,3]. These defects consist of a heterogenous group of structural heart malformations (i.e., conotruncal heart defects that affect the cardiac outflow tract) that are thought to have at least some shared genetic basis [4,5,6,7]. Some conotruncal heart defects involve a deviation from the normal position of the origin of the aorta and pulmonary trunk, in which case the great vessels are said to be transposed. Normally related great vessels indicate that the aorta emerges from the left ventricle while the pulmonary artery arises from the right ventricle. Conotruncal heart defects with normally related great vessels (CTD-NRGVs) frequently occur in individuals with a hemizygous 22q11.2 deletion, whereas those with transposed great vessels very rarely occur in the context of a 22q11.2 deletion. This suggests that the genetic basis of CTD-NRGV may differ from CTDs with transposed vessels. Further, since CTD-NRGVs occur both in individuals with and without a 22q11.2 deletion, there may be overlap in the genetic contribution to CTD-NRGV in these groups. This potential overlap in genetic etiology could include genetic variation within as well as outside of the 22q11.2 region, though this hypothesis has not been extensively studied. 

Both common copy number variants and common single nucleotide polymorphisms (SNPs) have been found to be associated with increased risk for heart defects in individuals with the 22q11.2DS [8]. Further, common variants in the distal region of the remaining 22q11.2 allele have been associated with increased risk for these defects among individuals with 22q11.2DS [6]. These data suggest that genetic variation within and outside of the deleted region contributes to the risk of heart defects in individuals with the deletion. In addition, association studies of rare copy number variants (rCNVs) suggest that at least some overlap in the genes and pathways that are involved in CTD-NRGVs in patients with and without 22q11.2DS [9,10,11]. For example, among separate cohorts of individuals with and without 22q11.2DS, Xie et al. (2019) identified 14 gene sets from Reactome pathways of interest [12] (e.g., gene silencing by RNA pathway, TGF-beta signaling pathway), with rCNVs over-represented among patients with CTD-NRGVs compared to controls without heart defects [10].

In general, both common and rare inherited variants are thought to play a role in conotruncal defects [7,13,14]. However, prior genome-wide association studies (GWAS) of conotruncal heart defects, both among cases with and without 22q11.2DS, have had somewhat limited success in identifying significant associations. Most of these initial studies have been limited by a fairly small number of cases, and the subset of conotruncal defects with NRGVs has not been evaluated in cases which do not have 22q11.2DS.

To assess the possibility of shared genetic susceptibility to CTD-NRGV between those with and those without a 22q11.2 deletion, we conducted GWAS and meta-analyses at the SNP-level and conducted gene-level GWAS, as well as gene set analyses using the rCNV Reactome pathways identified by Xie et al. [10]. 

## 2. Materials and Methods

### 2.1. Study Subjects

#### 2.1.1. Subjects without 22q11.2DS

**Figure 1:**
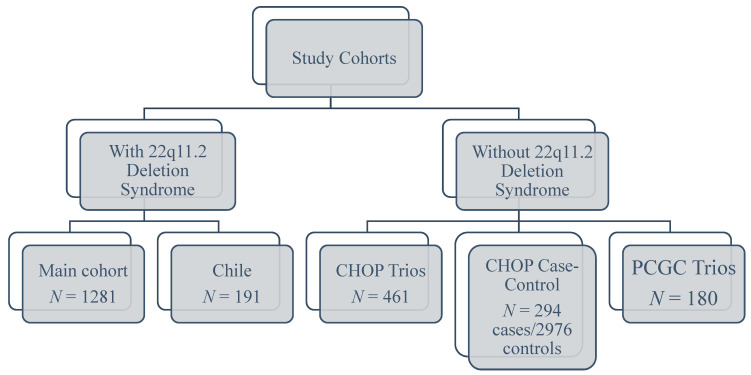
Summary of congenital heart defects cohorts. CHOP, The Children’s Hospital of Philadelphia; PCGC, The Pediatric Cardiac Genomics Consortium.

CTD-NRGVs were defined based on the presence of normally related great vessels in the context of at least one of the following diagnoses: tetralogy of Fallot, ventricular septal defects (conoventricular, posterior malalignment, and conoseptal hypoplasia), isolated aortic arch anomalies, truncus arteriosus, and interrupted aortic arch. Normally related great vessels are defined by the association of the pulmonary artery with the right ventricle and the aorta with the left ventricle (i.e., the presence of fibrous continuity between the aortic and mitral valves), where the aortic valve is situated posteriorly and just rightward of the pulmonary valve. Participants with CTD-NRGVs, but without documented 22q11.2 deletions, and their parents were recruited at the Children’s Hospital of Philadelphia (CHOP) during 1999–2010 and through the Pediatric Cardiac Genomics Consortium (PCGC) during 2010–2012, as previously described [13] (Figure 1). In both CHOP and PCGC groups, cases with suspected syndromes, including 22q11.2DS, were excluded. Further, all CHOP cases screened negative for a 22q11.2 deletion, using fluorescence in situ hybridization and/or multiplex ligation-dependent probe amplification [15,16]. Potential cases with other documented genetic syndromes were also excluded, based on a review of cardiac medical records [13]. To allow for case–control analyses among cases without trio data (e.g., missing parent samples), data for pediatric controls undergoing well-child visits at CHOP were also obtained [13]. Because trio-based analyses are robust to potential population stratification [17], trios of any race/ethnicity were included. However, all cases and controls were self-reported Caucasians, as case–control analyses are more sensitive to this potential bias. Each participant or parent provided informed consent under protocols approved by the institutional review boards at CHOP or the PCGC clinical study sites.

#### 2.1.2. Subjects with 22q11.2DS

Data for subjects with 22q11.2DS were obtained from affected subjects and their parents recruited by the International Chromosome 22q11.2 Deletion Syndrome Consortium, the International 22q11.2 Brain Behavior Consortium, and clinical groups that specialize in the treatment of individuals with 22q11.2DS, as previously described [18] (Figure 1). For all cases, the 22q11.2 deletion was confirmed using fluorescence in situ hybridization and/or multiplex ligation-dependent probe amplification [18]. Subjects with CTD-NRGVs were considered to be “cases” and those without a clinically significant heart defect were considered to be “controls.” Of note, a substantial proportion of subjects were recruited in Santiago, Chile, and this cohort was genotyped and analyzed separately [18]. Each participant or parent provided informed consent under protocols approved by the institutional review board at Albert Einstein College of Medicine.

### 2.2. Genotyping, Quality Control, and Prior Analyses

#### 2.2.1. Subjects without 22q11.2DS

Genomic DNA was genotyped using Illumina arrays and additional genotypes were imputed using reference data from the 1000 Genomes Project, as previously described [13]. Pre-imputation quality control measures included exclusion of case–parent trios (Mendelian error rate > 1%) and variants with minor allele frequency < 1% or genotyping rate < 90%. Post-imputation quality control measures included exclusion of variants with minor allele frequency < 5%, genotyping rate < 90%, or *r*^2^ < 0.8, which suggests poor imputation. At that stage, we also excluded variants and individuals with genotyping rates < 90%.

We have previously described SNP- [13] and gene-level [19] GWAS of a broader group with any conotruncal defects. The present analysis involved only the subset of those cases with CTD-NRGVs, a group we have not previously reported on.

#### 2.2.2. Subjects with 22q11.2DS

Genomic DNA was genotyped using an Affymetrix array and additional genotypes were imputed using reference data from the 1000 Genomes Project, as previously described [18]. Pre-imputation quality control measures included exclusion of variants with minor allele frequency < 1%, genotyping rate < 95%, or deviation from Hardy–Weinberg equilibrium in controls based on *p* ≤ 1 × 10^−5^. Post-imputation quality control measures included the exclusion of variants with minor allele frequency ≤ 1%, or *r*^2^ < 0.8.

We have previously conducted a GWAS of cases with 22q11.2 deletions and one specific conotruncal defect, tetralogy of Fallot [18]. The present analysis involved these subjects as well as the broader group of subjects with any CTD-NRGVs, for which we have not previously reported results. 

### 2.3. Statistical Methods

#### 2.3.1. SNP-Level Analyses

Separate SNP-level analyses were conducted for five individual cohorts, including three without a 22q11.2 deletion (461 CHOP trios, 180 PCGC trios, 294 CHOP cases/2976 CHOP controls) and two with 22q11.2DS (191 Chilean subjects with arrays processed in Santiago, Chile and 1281 subjects in the main cohort, 1244 with arrays processed at Albert Einstein College of Medicine, and 37 with arrays processed at the Children’s Research Institute in Milwaukee, WI, USA), as previously described [13,18]. For the cases without a 22q11.2 deletion, 29% had tetralogy of Fallot and 71% had other defects; however, for the subjects with 22q11.2DS, 22% were cases with tetralogy of Fallot, 39% were cases with other defects, and 38% were controls without a clinically significant congenital heart defect. Briefly, trios were analyzed using a multinomial likelihood approach [20] implemented in the EMIM software package [21], and the case–control analyses were conducted using logistic regression based on an additive genetic risk model and adjusted for principle components of race/ethnicity (the first four components for the cohorts with 22q11.2DS and the first two components for the cohorts without 22q11.2DS). Because subjects with 22q11.2DS are hemizygous for all loci within the 1.5–3 million base-pair deleted region, we excluded genes in this region in the analyses of the cohorts with a 22q11.2 deletion and in the meta-analysis of all five cohorts. Following these five cohort-specific analyses, we conducted three meta-analyses using GWAMA v2.1 [22], restricted to variants that were present across all five cohorts (with the exception of the variants in the 22q11.2 hemizygous deletion region). These included analyses of individuals with 22q11.2DS and those without 22q11.2DS (all five cohorts), as well as separate meta-analyses for individuals without 22q11.2DS (three cohorts) and individuals with 22q11.2DS (two cohorts). We used a fixed-effects model for these analyses when Cochran’s heterogeneity *p* > 0.1, and a random-effects model when Cochran’s heterogeneity *p* ≤ 0.1 [13].

#### 2.3.2. Gene-Level Analyses

Using MAGMA version 1.08 [23], gene-level analyses were conducted using SNP-level summary statistics from each meta-analysis as input. SNPs were annotated to protein-coding genes, defined by their transcription start–stop coordinates, using NCBI 37.3 (downloaded from https://ctg.cncr.nl/software/magma, accessed on 6 June 2019). SNPs within 1 kb upstream or downstream of the start or stop coordinates were included in the annotation window and also mapped to the gene.

Gene-level *p*-values were calculated from the SNP-level summary statistics for each meta-analysis. Magma software can estimate the gene-level *p*-value by using the mean test statistic for the SNPs or the top test statistic among the SNPs. Magma can also estimate an aggregate *p*-value obtained by combining both test statistics. For our analyses, we used the aggregate statistic to ensure even distribution of power and to account for a wider range of genetic models. The computed gene-level *p*-values were transformed to a Z-score using the probit transformation, with lower *p*-values (i.e., more significant associations) being associated with higher Z-scores. These Z-scores served as input for the gene set analyses.

#### 2.3.3. Candidate Gene Set Analyses

We used MAGMA to conduct candidate gene set association analyses. First, we evaluated the 42 genes in the 22q11.2DS 3 Mb region as a single gene set among the non-deleted cohorts. Second, we separately evaluated 14 Reactome pathways identified in the rCNV study reported by Xie et al. [10]. This group of genes represents statistically significant shared pathways, expression patterns, and biological functions between patients with versus without conotruncal heart defects among patients with and without 22q11.2DS [10]. We also evaluated an additional aggregate gene set consisting of genes present in any of these 14 gene sets.

The gene-level association results for each gene were used as the input for these gene set association analyses. Specifically, each gene *p*-value computed from the gene-level association analysis was converted to a Z-score, which served as the dependent variable [23]. For these comparisons, we used competitive (as opposed to self-contained) association tests under a linear regression framework, which evaluate whether the genes in the set of interest are more strongly associated with a phenotype as compared to all other genes in the genome (i.e., β_s _= 0 against the alternative hypothesis β_s _> 0), correcting for gene size, gene density, differential sample size, and the log of those values [23]. This analysis corrects for potential confounders including gene size, density, and sample size by adding these variables and their log as additional covariates in the gene-level regression model. To adjust for linkage disequilibrium between genes, a gene–gene correlation matrix was approximated and included in the model (for gene pairs over 5 Mb apart, the correlation was set to zero) [23]. 

#### 2.3.4. Interpretation

For the SNP-level analyses, we used the standard GWAS threshold (*p* < 5.0 × 10^−8^) to identify statistically significant associations. SNP associations with *p* ≥ 5.0 × 10^−8^ but less than *p* < 1.0 × 10^−5^ were considered suggestive of association. For the gene and gene set analyses, we used a Bonferroni correction for the total number of genes and gene sets, respectively. Genes associated with *p* < 1.0 × 10^−3^ but greater than the Bonferroni-corrected cut-off were considered to be suggestive of association.

## 3. Results

### 3.1. SNP-Level

SNP-level analyses were conducted separately for the five individual cohorts (N = 3,311,160 SNPs). No SNP association achieved genome-wide significance (*p* < 5.0 × 10^−8^) in any of the three meta-analyses (Tables S1 and S2). The smallest *p*-value was 1.6 × 10^−7^ (rs6886261 in the non-deleted cohort) and a number of SNPs had *p*-values suggestive of association (*p* < 1.0 × 10^−5^) (12 SNPs among individuals with 22q11.2DS, 147 among individuals without 22q11.2DS, and 129 among individuals with 22q11.2DS + without 22q11.2DS). However, no SNP association was suggestive of association in both meta-analysis of individuals with 22q11.2DS and meta-analysis of individuals without 22q11.2DS.

### 3.2. Gene-Level

For each of the three analytic groups (individuals with 22q11.2DS, without 22q11.2DS, and with 22q11.2DS + without 22q11.2DS), we conducted gene-level analyses (13,650 genes evaluated). No gene achieved genome-wide significance (*p* < 3.7 × 10^−6^) among these comparisons (Table S3). Although 43 genes had *p*-values < 1.0 × 10^−3^, none were suggestive of association in both the comparison among individuals with 22q11.2DS and the comparison among individuals without 22q11.2DS. The lowest *p*-values were 3.2 × 10^−5^ for *TBC1D21* among individuals without 22q11.2DS and 5.6 × 10^−5^ for *GLYATL3* among individuals with 22q11.2DS. 

In the comparison of individuals with 22q11.2DS + without 22q11.2DS, 26 genes had suggestive evidence of association. Of these, eight (*EVX1, INPP4B, SLC35G5, ARHGAP26, DEFB134, CASQ2, PTCH1,* and *STAB2)* also had *p*-values < 0.05 in both the comparison among individuals with 22q11.2DS and the comparison among individuals without 22q11.2DS (*p*-value range: 8.3 × 10^−5^ to 8.5 × 10^−4^) (Table 1).

Among the 42 genes in the 22q11.2DS 3 Mb region, 5 had *p*-values < 0.05 (Table S4) among individuals without 22q11.2DS, the lowest being *HIRA* (*p* = 0.03).

### 3.3. Gene Sets

Among individuals without 22q11.2DS only, the 42 genes in the 3 Mb 22q11.2 deleted interval were evaluated as a single gene set. However, this set was not significantly associated with CTD-NRGV in these data (*p* = 0.49). The 14 individual gene sets and aggregate gene set (all gene sets combined) from the rCNV Reactome pathways identified by Xie et al. [10] were assessed in all three groups (Table 2). No gene set was significantly associated with conotruncal defects with NRGVs after accounting for multiple comparisons using a Bonferroni correction for 14 comparisons (based on *p* < 3.6 × 10^−3^). The lowest gene set *p*-values included 6.6 × 10^−3^ (Gene Silencing by RNA gene set among individuals without 22q11.2DS) and 5.6 × 10^−3^ (ECM–receptor interaction gene set among individuals with 22q11.2DS).

## 4. Discussion

Our findings from genome-wide SNP- and gene-level analyses and candidate gene set analyses among these cohorts did not provide strong evidence for associations due to common variants in either cohort or in the combined cohorts. Thus, while we did not observe results that strongly supported the hypothesis that there are shared genomic mechanisms involving common inherited variants for CTD-NRGVs across subjects with and without 22q11.2DS, our results also did not refute this hypothesis. Gene and gene set analyses among the 3 Mb 22q11.2 region and analyses of gene sets from the rCNV gene interaction network [10] did not strongly support or refute the notion that the respective regions may contribute to conotruncal defects with CTD-NRGVs among both deleted and non-deleted cases. Nevertheless, several results were suggestive of association, even in the absence of achieving statistical significance, and may represent helpful candidates to consider further in future work. 

We found some suggestive evidence for association between SNPs and CTD-NRGVs, particularly among the cohorts without 22q11.2DS. Of the 129 SNPs with *p* < 1.0 × 10^−5^ among the comparison of individuals with 22q11.2DS + without 22q11.2DS, 33 corresponded to *INPP4B*, and the majority of these SNPs also had *p* < 0.05 in both the separate comparison of individuals with 22q11.2DS and comparison of individuals without 22q11.2DS. In fact, similar trends were also observed for *INPP4B* among the gene-level comparisons, and it was the gene with the second-lowest *p*-value in Table 1. *INPP4B* is a Mg(2+)-independent phosphatase that is highly expressed in the heart [24], and it is a tumor suppressor involved in the inhibition of PI3K signaling [25]. However, relatively little is known about the function of this gene, and it is unclear what, if any, role this gene may play in cardiogenesis. Additionally, 48 of the 129 SNPs with suggestive associations in the comparison of individuals with 22q11.2DS + without 22q11.2DS corresponded to *TULP4*, a candidate gene for craniofacial cleft and short stature that has also been implicated as a contributing gene in a patient with features of 22q11.2DS but without a 22q11.2 deletion [26]. However, suggestive associations with these SNPs in *TULP4* were actually observed in our comparison of individuals without 22q11.2DS, but not in our comparison of individuals with 22q11.2DS. Similar trends were observed for *TULP4* among our gene-level comparisons.

Several of the other top genes from our gene set analyses that are thought to be related to cardiogenesis but did not achieve genome-wide significance may still represent good candidates for CTD-NRGVs, either among individuals with or without 22q11.2DS. For example, including *INPP4B*, 8 of the 26 suggestive genes had a *p*-value < 0.05 in both individual cohorts as well as a lower *p*-value in the combined cohort than in either individual cohort. Of these eight genes, a potential candidate for future work is *EVX1*, which is an agonist of cardiogenic mesoderm formation [27]. Another, *PTCH1,* is involved in TGF-beta, Wnt, and SHH signaling, which are all thought to be involved in secondary heart field development [28]. Specifically, *PTCH1* encodes the main receptor for sonic hedgehog, which is required for normal development of the cardiac outflow tract, and SHH signaling is a major candidate pathway for CTD-NRGVs, both in patients with and without 22q11.2DS [29]. Heart abnormalities and open neural tube defects are also present among mice with homozygous *Ptch1* mutations, which are embryonically lethal [30].

Among the 14 gene sets from the rCNV Reactome pathways identified by Xie et al. [10], there were associations between the Gene Silencing by RNA gene set and CTD-NRGVs without 22q11.2DS, as well as between the ECM–receptor interaction gene set and CTD-NRGVs with 22q11.2DS, though these associations would not be significant after considering a Bonferroni correction for the number of gene set comparisons. Disruption of genes involved in the composition and remodeling of the extracellular matrix (ECM) of the developing heart can result in cardiac malformations [31]. Although no gene sets had *p* < 0.05 for both the comparison of individuals with 22q11.2DS and the comparison of individuals without 22q11.2DS, the *p*-value for all pathways combined in the comparison among individuals with 22q11.2DS + without 22q11.2DS was low (*p* = 0.016), as well as smaller than that from the other two separate comparisons. This may provide some suggestive further evidence of genetic overlap between the etiologies of these defects that involves not only rCNVs [10] but also more common genetic variation.

Though our analyses did not detect strong evidence for genetic similarities between deleted and non-deleted conotruncal defects with CTD-NRGVs, it may be that our sample was not sufficiently powered to detect modest associations. Our restriction to variants present in all five cohorts (i.e., for variants outside of the 3 Mb 22q11.2 region) also may have resulted in the elimination of SNPs or genes than could have been associated within subsets of the five cohorts. Further, if there are heterogeneous genetic effects between subtypes of conotruncal defects with CTD-NRGVs, more homogeneous subgroups may be more helpful to focus on in future work (e.g., tetralogy of fallot), though sub-group analyses were beyond the scope of these analyses and require larger samples. Similar to other genome-wide studies, we conducted a number of comparisons and used a Bonferroni correction within but not across each analytic group.

Strengths of this study include access to data from individuals with and without 22q11.2 deletions, and the use of case–parent trio samples, which allow for analyses that do not require external controls and are robust to potential bias related to population stratification.

## 5. Conclusions

In summary, we report on a number of potential candidate regions for CTD-NRGV, both among individuals with and without 22q11.2DS. We did not observe strong evidence of overlap in associations involving common variants between these two groups, and more work is needed to evaluate other forms of genomic variation, as well as phenotypic subgroups.

## Figures and Tables

**Table 1:** Summary data for genes with *p* < 1.0 × 10^−3^, by cohort ^1,2^.

Gene Name (Gene Symbol)	Chr ^3^	Start ^4^	Stop	Cohort with 22q.11.2 Deletion Syndrome	Cohort without a 22q.11.2 Deletion	All Cohorts Combined
*Even-Skipped Homeobox 1 (EVX1)*	7	27281164	27288438	2.40 × 10^−3^	4.20 × 10^−2^	8.33 × 10^−5^
*Inositol Polyphosphate-4-Phosphatase Type II B (INPP4B)*	4	142948181	143768604	1.60 × 10^−2^	4.40 × 10^−2^	5.00 × 10^−4^
*Solute Carrier Family 35 Member G5 (SLC35G5)*	8	11187495	11190695	4.30 × 10^−2^	4.60 × 10^−3^	5.00 × 10^−4^
*Rho GTPase Activating Protein 26 (ARHGAP26)*	5	142148881	142609572	4.60 × 10^−2^	1.70 × 10^−2^	6.00 × 10^−4^
*Defensin Beta 134 (DEFB134)*	8	11850489	11854760	1.10 × 10^−2^	4.70 × 10^−2^	6.00 × 10^−4^
*Calsequestrin 2 (CASQ2)*	1	116241624	116312426	1.90 × 10^−2^	4.70 × 10^−2^	6.50 × 10^−4^
*Patched 1 (PTCH1)*	9	98204264	98280247	3.10 × 10^−2^	1.10 × 10^−2^	7.00 × 10^−4^
*Stabilin 2 (STAB2)*	12	103980069	104161502	2.50 × 10^−2^	1.12 × 10^−2^	8.50 × 10^−4^

^1^ Data only shown for genes with *p*-values < 0.05 in both individual cohorts (deleted, non-deleted) and with a lower *p*-value in the combined (deleted + non-deleted) cohort than in both individual cohorts. ^2^ Three meta-analyses: cohort with 22q.11.2 deletion syndrome; cohort without 22q.11.2 deletion syndrome; all cohorts combined. ^3^ Chromosome. ^4^ Genome Reference Consortium Human genome build 37/hg19 reference assembly.

**Table 2:** Gene set analysis results for 14 gene sets from the rCNV-derived unique gene networks (including all gene sets combined).

		Cohort with 22q.11.2 Deletion Syndrome	Cohort without 22q.11.2 Deletion Syndrome	All Cohorts Combined
Gene Set	Number of Genes	*p*-Value	*p*-Value	*p*-Value
All gene sets combined	250	0.274	0.333	0.016
Gene Silencing by RNA	6	0.453	0.007	0.007
Integrin signaling pathway	29	0.067	0.417	0.288
TGF-beta signaling pathway	22	0.358	0.148	0.085
ECM-receptor interaction	16	0.006	0.075	0.050
Regulation of mitotic cell cycle	10	0.840	0.736	0.191
G alpha (q) signaling events	26	0.036	0.649	0.230
Basal transcription factors	6	0.044	0.670	0.134
Cardiac conduction	20	0.266	0.773	0.253
Mitochondrial translation	4	0.923	0.187	0.448
Mitotic Prometaphase	34	0.757	0.352	0.399
Processing of Capped Intron-Containing Pre-mRNA	27	0.889	0.376	0.409
MAPK signaling pathway	40	0.915	0.648	0.516
Neddylation	6	0.774	0.877	0.794
Chromatin organization	4	0.327	0.987	0.983

## Data Availability

The genotype data used in these studies are available at: Pediatric Cardiac Genomics Consortium: https://www.ncbi.nlm.nih.gov/projects/gap/cgi-bin/study.cgi?study_id=phs001194.v2.p2CHOPCTDs; https://www.ncbi.nlm.nih.gov/projects/gap/cgi-bin/study.cgi?study_id=phs000881.v1.p1CHOPpediatriccontrols; https://www.ncbi.nlm.nih.gov/projects/gap/cgi-bin/study.cgi?study_id=phs000490.v1.p122q11.2DScohorts; https://www.ncbi.nlm.nih.gov/projects/gap/cgi-bin/study.cgi?study_id=phs001339.v1.p1.

## Supplementary Materials

The following are available online at https://www.mdpi.com/article/10.3390/genes12071030/s1, Table S1: Summary data for genes and SNPs with SNP *p* < 1.0 × 10^−5^ in the combined cohort and SNP *p* < 0.05 in both individual cohorts, Table S2: SNPs with suggestive evidence of association (*p* < 1.0 × 10^−5^) in the three meta-analyses, Table S3: Genes with suggestive evidence of association (*p* < 1.0 × 10^−3^), Table S4: Summary data for genes located in the 22q.11.2 deletion region (chr22:14,700,001-25,900,000) among the cohort without 22q11.2DS, sorted by *p*-value.

## References

[1] Howell H.B., Zaccario M., Kazmi S.H., Desai P., Sklamberg F.E., Mally P. (2019). Neurodevelopmental outcomes of children with congenital heart disease: A review. Curr. Probl. Pediatr. Adolesc. Health Care.

[2] Shabana N.A., Shahid S.U., Irfan U. (2020). Genetic Contribution to Congenital Heart Disease (CHD). Pediatr. Cardiol..

[3] Kirby R.S. (2017). The prevalence of selected major birth defects in the United States. Semin. Perinatol..

[4] Lahiri S., Gil W., Daria S., Joshua G., Parul J., Redmond B., Elizabeth W. (2020). Genetic abnormalities/syndromes significantly impact perioperative outcomes of conotruncal heart defects. Ann. Pediatr. Cardiol..

[5] DeLea M., Espeche L.D., Bruque C.D., Bidondo M.P., Massara L.S., Oliveri J., Brun P., Cosentino V.R., Martinoli C., Tolaba N. (2018). Genetic Imbalances in Argentinean Patients with Congenital Conotruncal Heart Defects. Genes.

[6] Zhao Y., Diacou A., Johnston H.R., Musfee F.I., McDonald-McGinn D.M., McGinn D., Crowley T.B., Repetto G.M., Swillen A., Breckpot J. (2020). Complete Sequence of the 22q11.2 Allele in 1,053 Subjects with 22q11.2 Deletion Syndrome Reveals Modifiers of Conotruncal Heart Defects. Am. J. Hum. Genet..

[7] Lyu C., Webber D.M., MacLeod S.L., Hobbs C.A., Li M., the National Birth Defects Prevention Study (2019). Gene-by-gene interactions associated with the risk of conotruncal heart defects. Mol. Genet. Genom. Med..

[8] Mlynarski E.E., Sheridan M.B., Xie M., Guo T., Racedo S.E., McDonald-McGinn D.M., Gai X., Chow E.W., Vorstman J., Swillen A. (2015). Copy-Number Variation of the Glucose Transporter Gene SLC2A3 and Congenital Heart Defects in the 22q11.2 Deletion Syndrome. Am. J. Hum. Genet..

[9] Mlynarski E.E., Xie M., Taylor D., Sheridan M.B., Guo T., Racedo S.E., McDonald-McGinn D.M., Chow E.W.C., Vorstman J., Swillen A. (2016). Rare copy number variants and congenital heart defects in the 22q11.2 deletion syndrome. Qual. Life Res..

[10] Xie H.M., Taylor D.M., Zhang Z., McDonald-McGinn D.M., Zackai E.H., Stambolian D., Hakonarson H., Morrow B.E., Emanuel B.S., Goldmuntz E. (2019). Copy number variations in individuals with conotruncal heart defects reveal some shared developmental pathways irrespective of 22q11.2 deletion status. Birth Defects Res..

[11] Xie H.M., Werner P., Stambolian D., Bailey-Wilson J.E., Hakonarson H., White P.S., Taylor D.M., Goldmuntz E. (2017). Rare copy number variants in patients with congenital conotruncal heart defects. Birth Defects Res..

[12] Jassal B., Matthews L., Viteri G., Gong C., Lorente P., Fabregat A., Sidiropoulos K., Cook J., Gillespie M., Haw R. (2020). The reactome pathway knowledgebase. Nucleic Acids Res..

[13] Agopian A.J., Goldmuntz E., Hakonarson H., Sewda A., Taylor D., Mitchell L.E., Pediatric Cardiac Genomics Consortium (2017). Genome-Wide Association Studies and Meta-Analyses for Congenital Heart Defects. Circ. Cardiovasc. Genet..

[14] Sewda A., Agopian A.J., Goldmuntz E., Hakonarson H., Morrow B.E., Musfee F., Taylor D., Mitchell L.E., Pediatric Cardiac Genomics Consortium (2020). Gene-based analyses of the maternal genome implicate maternal effect genes as risk factors for conotruncal heart defects. PLoS ONE.

[15] Goldmuntz E., Clark B.J., Mitchell L.E., Jawad A.F., Cuneo B.F., Reed L., McDonald-McGinn D., Chien P., Feuer J., Zackai E.H. (1998). Frequency of 22q11 deletions in patients with conotruncal defects. J. Am. Coll. Cardiol..

[16] Peyvandi S., Lupo P.J., Garbarini J., Woyciechowski S., Edman S., Emanuel B.S., Mitchell L.E., Goldmuntz E. (2013). 22q11.2 Deletions in Patients with Conotruncal Defects: Data from 1,610 Consecutive Cases. Pediatr. Cardiol..

[17] Wilcox A., Weinberg C., Lie R.T. (1998). Distinguishing the Effects of Maternal and Offspring Genes through Studies of “Case-Parent Triads”. Am. J. Epidemiol..

[18] Guo T., Repetto G.M., McGinn D.M.M., Chung J.H., Nomaru H., Campbell C.L., Blonska A., Bassett A.S., Chow E.W., Mlynarski E.E. (2017). Genome-Wide Association Study to Find Modifiers for Tetralogy of Fallot in the 22q11.2 Deletion Syndrome Identifies Variants in the GPR98 Locus on 5q14.3. Circ. Cardiovasc. Genet..

[19] Sewda A., Agopian A.J., Goldmuntz E., Hakonarson H., Morrow B.E., Taylor D., Mitchell L.E., on behalf of the Pediatric Cardiac Genomics Consortium (2019). Gene-based genome-wide association studies and meta-analyses of conotruncal heart defects. PLoS ONE.

[20] Ainsworth H.F., Unwin J., Jamison D.L., Cordell H.J. (2011). Investigation of maternal effects, maternal-fetal interactions and parent-of-origin effects (imprinting), using mothers and their offspring. Genet. Epidemiol..

[21] Howey R., Cordell H.J. (2012). PREMIM and EMIM: Tools for estimation of maternal, imprinting and interaction effects using multinomial modelling. BMC Bioinform..

[22] Magi R., Morris A.P. (2010). GWAMA: Software for genome-wide association meta-analysis. BMC Bioinform..

[23] De Leeuw C.A., Mooij J.M., Heskes T., Posthuma D. (2015). MAGMA: Generalized Gene-Set Analysis of GWAS Data. PLoS Comput. Biol..

[24] Norris F.A., Atkins R.C., Majerus P.W. (1997). The cDNA cloning and characterization of inositol polyphosphate 4-phosphatase type II. Evidence for conserved alternative splicing in the 4-phosphatase family. J. Biol. Chem..

[25] Gewinner C., Wang Z.C., Richardson A., Teruya-Feldstein J., Etemadmoghadam D., Bowtell D., Barretina J., Lin W.M., Rameh L., Salmena L. (2009). Evidence that Inositol Polyphosphate 4-Phosphatase Type II is a Tumor Suppressor that Inhibits PI3K Signaling. Cancer Cell.

[26] Wu D., Chen Y., Chen Q., Wang G., Xu X., Peng A., Hao J., He J., Huang L., Dai J. (2019). Clinical presentation and genetic profiles of Chinese patients with velocardiofacial syndrome in a large referral centre. J. Genet..

[27] Cunningham T., Yu M.S., McKeithan W.L., Spiering S., Carrette F., Huang C.-T., Bushway P.J., Tierney M., Albini S., Giacca M. (2017). Id genes are essential for early heart formation. Genes Dev..

[28] Dyer L.A., Kirby M.L. (2009). The role of secondary heart field in cardiac development. Dev. Biol..

[29] Smoak I.W., Byrd N., Abu-Issa R., Goddeeris M., Anderson R., Morris J., Yamamura K., Klingensmith J., Meyers E. (2005). Sonic hedgehog is required for cardiac outflow tract and neural crest cell development. Dev. Biol..

[30] Cooper A.F., Yu K.P., Brueckner M., Brailey L.L., Johnson L., McGrath J.M., Bale A.E. (2005). Cardiac and CNS defects in a mouse with targeted disruption of suppressor of fused. Development.

[31] Lockhart M., Wirrig E., Phelps A., Wessels A. (2011). Extracellular matrix and heart development. Birth Defects Res. Part A Clin. Mol. Teratol..

