# Supplementary material for: Genome-Wide Association Studies of Conotruncal Heart Defects with Normally Related Great Vessels in the United States
Source: Genes (Basel). 2021 Jul 1;12(7):1030. doi: 10.3390/genes12071030 (PMC8306129; doi:10.3390/genes12071030)
Supplement: Supplementary file 1 [file genes-12-01030-s001.zip › genes-1219636-supplementary.pdf]

Supplemental Table S1: Summary data for genes and SNPs with SNP $p < 1.0 \times 10^{-5}$ in the combined cohort and SNP $p < 0.05$ in both individual cohorts.[*, †]

				SNP-level p-values			Gene-level p-value
**SNP**	**Gene Name**	**Chr: Position (bp)**	**Reference Allele**	**Cohort with 22q.11.2 deletion syndrome**	**Cohort without a 22q.11.2 deletion**	**All cohorts combined**	**All cohorts combined**
rs13102150	*INPP4B*	4:143470133	C	1.83E-04	7.37E-04	6.43E-07	5.00E-04
rs3843430	intragenic	4:14101273	T	3.33E-05	3.67E-03	1.16E-06	--
rs11100748	*INPP4B*	4:143460923	A	5.00E-04	9.02E-04	1.75E-06	5.00E-04
rs60280851	*CORO2B*	15:68959002	G	3.98E-02	9.75E-06	1.93E-06	2.00E-04
rs1923665	*NFASC*	1:204864152	A	1.22E-02	4.44E-05	2.00E-06	3.25E-04
rs12504375	INPP4B	4:143456800	C	6.86E-04	9.20E-04	2.34E-06	5.00E-04
rs34054994	*CORO2B*	15:68958238	G	4.02E-02	1.40E-05	2.66E-06	2.00E-04
rs4975317	*INPP4B*	4:143457845	T	8.03E-04	9.42E-04	2.76E-06	5.00E-04
rs6835296	*INPP4B*	4:143455287	A	1.33E-02	5.81E-05	2.76E-06	5.00E-04
rs911988	*NFASC*	1:204863306	T	1.24E-02	6.44E-05	2.85E-06	3.25E-04
rs2351027	*LOC105377369*	4:112790034	C	6.21E-03	1.44E-04	2.89E-06	--
rs11930912	*INPP4B*	4:143461480	T	5.73E-04	1.38E-03	3.18E-06	5.00E-04
rs17016344	*INPP4B*	4:143469061	G	2.12E-04	3.34E-03	3.55E-06	5.00E-04
rs58899027	*INPP4B*	4:143465318	A	1.59E-04	4.33E-03	3.81E-06	5.00E-04
rs4246729	*INPP4B*	4:143468371	G	1.99E-04	3.85E-03	3.99E-06	5.00E-04
rs4101198	intragenic	4:14044759	G	4.77E-04	1.89E-03	4.24E-06	--
rs6847349	intragenic	4:14040923	G	1.39E-04	4.36E-03	4.38E-06	--
rs1425518	*INPP4B*	4:143462437	C	1.04E-04	6.71E-03	4.56E-06	5.00E-04
rs1476122	*INPP4B*	4:143469236	G	2.12E-04	4.26E-03	4.71E-06	5.00E-04
rs3801321	intragenic	7:27279191	T	2.67E-04	3.39E-03	5.02E-06	--
rs4975306	*INPP4B*	4:143453881	A	1.37E-03	1.12E-03	5.27E-06	5.00E-04
rs2059511	*INPP4B*	4:143460080	A	5.78E-04	2.30E-03	5.41E-06	5.00E-04
rs1535478	intragenic	9:7990651	T	2.94E-05	1.55E-02	5.43E-06	--
rs4975313	*INPP4B*	4:143468733	G	2.06E-04	4.96E-03	5.49E-06	5.00E-04
rs79391134	*INPP4B*	4:143460250	T	6.46E-04	2.13E-03	5.50E-06	5.00E-04
rs6816875	*INPP4B*	4:143464033	T	1.33E-04	6.70E-03	5.56E-06	5.00E-04
rs11100749	*INPP4B*	4:143461463	G	6.35E-04	2.26E-03	5.78E-06	5.00E-04

rs2059510	*INPP4B*	4:143459907	C	6.34E-04	2.30E-03	5.84E-06	5.00E-04
rs2217016	*INPP4B*	4:143464619	G	1.40E-04	6.81E-03	5.91E-06	5.00E-04
rs2059513	*INPP4B*	4:143460462	G	5.62E-04	2.59E-03	6.01E-06	5.00E-04
rs7671198	*INPP4B*	4:143470929	T	2.58E-04	4.69E-03	6.17E-06	5.00E-04
rs16998558	*INPP4B*	4:143471217	T	2.68E-04	4.65E-03	6.28E-06	5.00E-04
rs17016354	*INPP4B*	4:143472696	T	2.68E-04	4.75E-03	6.43E-06	5.00E-04
rs17717651	*INPP4B*	4:143453079	A	1.35E-03	1.37E-03	6.45E-06	5.00E-04
rs4975318	*INPP4B*	4:143454105	C	1.20E-03	1.54E-03	6.54E-06	5.00E-04
rs12504770	*INPP4B*	4:143452719	T	1.36E-03	1.40E-03	6.64E-06	5.00E-04
rs10900429	*NFASC*	1:204860968	G	1.03E-02	2.16E-04	7.06E-06	3.25E-04
rs7676337	intragenic	4:14038869	G	2.59E-04	4.41E-03	7.13E-06	--
rs6537109	*INPP4B*	4:143473479	A	4.02E-04	3.88E-03	7.33E-06	5.00E-04
rs12405099	*NFASC*	1:204861083	C	1.02E-02	2.27E-04	7.37E-06	3.25E-04
rs3846368	intragenic	4:14056459	G	3.54E-04	3.80E-03	7.47E-06	--
rs1373381	intragenic	4:14057145	T	3.54E-04	3.80E-03	7.48E-06	--
rs3857159	intragenic	4:14056930	T	3.54E-04	3.80E-03	7.48E-06	--
rs4975316	*INPP4B*	4:143457985	A	9.24E-04	2.18E-03	7.60E-06	5.00E-04
rs3846367	intragenic	4:14056393	T	3.32E-04	4.03E-03	7.65E-06	--
rs13221828	intragenic	7:27271127	C	2.95E-04	4.98E-03	8.27E-06	--
rs61104737	*INPP4B*	4:143455438	C	9.15E-04	2.45E-03	8.54E-06	5.00E-04
rs12504378	*INPP4B*	4:143456826	C	8.01E-04	2.74E-03	8.63E-06	5.00E-04
rs60951295	*INPP4B*	4:143475473	C	1.52E-04	7.95E-03	8.75E-06	5.00E-04
rs10519649	*INPP4B*	4:143454553	C	1.14E-03	2.13E-03	8.78E-06	5.00E-04
rs3906860	intragenic	4:14054875	T	3.87E-04	4.17E-03	8.92E-06	--
rs12405378	*NFASC*	1:204861371	C	1.02E-02	2.82E-04	9.02E-06	3.25E-04
rs7122295	intragenic	11:59050845	G	1.22E-02	2.50E-04	9.14E-06	--
rs11244263	*LAMC3*	9:133931123	C	4.65E-02	4.83E-03	9.21E-06	--
rs6827084	intragenic	4:14058506	T	3.64E-04	4.50E-03	9.28E-06	--
rs4484375	*INPP4B*	4:143450615	C	1.01E-02	3.05E-04	9.52E-06	5.00E-04
rs12405381	*NFASC*	1:204861384	C	1.02E-02	3.02E-04	9.60E-06	3.25E-04
rs3944007	intragenic	16:49004284	G	5.62E-03	5.57E-04	9.69E-06	--
rs4785155	intragenic	16:49003778	C	5.15E-03	6.08E-04	9.72E-06	--

* Data only shown for SNPs with p-values < 0.05 in both individual cohorts and with $p < 1.0 \times 10^{-5}$ (suggestive p-values) in the combined cohort

† Three meta-analyses: cohort with 22q.11.2 deletion syndrome; cohort without 22q.11.2 deletion syndrome; all cohorts combined

Supplemental Table S2: SNPs with suggestive evidence of association ($p<1.0 \times 10^{-5}$) in the three meta-analyses.

SNP	Chr: Position (bp)	Reference Allele	Gene	Cohort with 22q.11.2 deletion syndrome meta-analysis	Cohort without 22.q.11.2 deletion syndrome meta-analysis	All cohorts meta-analysis
**Cohort with 22q.11.2 deletion syndrome**						
rs7720206	5: 90068209	C	*ADGRV1*	**1.35E-06**	8.91E-01	4.26E-03
rs12519770	5: 90073277	A	*ADGRV1*	**1.68E-06**	8.72E-01	4.81E-03
rs13188479	5: 90070979	A	*ADGRV1*	**3.50E-06**	8.31E-01	7.13E-03
rs9381793	6: 49484201	G	*GLYATL3*	**4.39E-06**	1.93E-01	1.20E-04
rs10133981	14: 77735793	G	*NGB*	**5.10E-06**	9.10E-01	8.74E-03
rs75580157	4: 167913696	T	intragenic	**5.16E-06**	9.51E-01	7.48E-03
rs7660050	4: 167959369	C	*SPOCK3*	**6.80E-06**	6.76E-01	2.01E-02
rs6553490	4: 167933010	A	*SPOCK3*	**8.16E-06**	7.02E-01	3.36E-03
rs2443079	5: 90110224	A	*ADGRV1*	**8.61E-06**	9.90E-01	6.34E-03
rs1193773	11: 63019141	T	*LOC105369334*	**8.62E-06**	6.98E-02	1.60E-01
rs2438368	5: 90094855	G	*ADGRV1*	**8.94E-06**	9.18E-01	5.04E-03
rs9395502	6: 49484646	G	*GLYATL3*	**9.93E-06**	1.85E-01	1.85E-04
**Cohort without a 22q.11.2 deletion**						
rs6886261	5:43022764	C	intragenic	2.97E-01	**1.58E-07**	1.27E-04
rs921541	15:74160103	G	*LOC102723657*	2.10E-01	**1.65E-07**	9.28E-07
rs13353396	3:34547165	C	intragenic	3.16E-01	**2.61E-07**	2.95E-06
rs585302	5:43034142	T	intragenic	2.96E-01	**3.49E-07**	1.83E-04
rs7163502	15:74164031	T	intragenic	4.73E-01	**3.55E-07**	4.09E-04
rs4886717	15:74165128	C	intragenic	3.61E-01	**4.64E-07**	6.76E-04
rs2108975	17:11221167	C	*SHISA6*	3.81E-01	**5.70E-07**	7.21E-04
rs13411840	2:29973281	C	*ALK*	4.72E-01	**5.86E-07**	3.87E-04
rs839156	12:86595690	A	*MGAT4C*	5.40E-01	**6.02E-07**	4.25E-04
rs7178188	15:74153876	G	intragenic	1.69E-01	**6.15E-07**	1.12E-03
rs1852808	3:34556501	T	intragenic	2.45E-01	**7.04E-07**	3.89E-06

rs78977294	8:117409142	T	intragenic	9.69E-01	**7.92E-07**	1.59E-04
rs2813392	10:1628905	C	*ADARB2*	4.74E-01	**7.95E-07**	3.95E-04
rs863394	12:86594783	A	*MGAT4C*	5.47E-01	**8.63E-07**	5.12E-04
rs6465387	7:93154060	G	*CALCR*	3.17E-02	**9.65E-07**	1.74E-02
rs839152	12:86597000	A	*MGAT4C*	4.77E-01	**1.09E-06**	7.79E-04
rs11012462	10:21275353	A	*NEBL*	6.41E-01	**1.37E-06**	2.83E-04
rs55804929	8:117409861	T	intragenic	8.94E-01	**1.39E-06**	1.60E-04
rs6882380	5:42921701	A	intragenic	2.28E-01	**1.41E-06**	9.14E-04
rs6996802	8:58293378	C	intragenic	3.00E-01	**1.49E-06**	5.13E-06
rs7018443	8:58293505	G	intragenic	3.02E-01	**1.49E-06**	5.21E-06
rs865721	12:86589761	T	*MGAT4C*	6.13E-01	**1.61E-06**	6.73E-04
rs12942682	17:11224231	G	*SHISA6*	3.85E-01	**1.62E-06**	1.17E-03
rs10071470	5:42921929	G	intragenic	1.92E-01	**1.66E-06**	9.99E-04
rs12943640	17:11224534	G	*SHISA6*	3.82E-01	**1.66E-06**	1.21E-03
rs6996833	8:58293434	C	intragenic	3.00E-01	**1.77E-06**	5.96E-06
rs6982211	8:58294313	T	intragenic	3.51E-01	**1.82E-06**	8.14E-06
rs55960888	8:58294130	G	intragenic	3.09E-01	**1.83E-06**	6.42E-06
rs6981267	8:58293977	G	intragenic	3.09E-01	**1.83E-06**	6.41E-06
rs6981437	8:58294054	G	intragenic	3.09E-01	**1.83E-06**	6.41E-06
rs6981568	8:58293936	A	intragenic	3.09E-01	**1.83E-06**	6.41E-06
rs6985947	8:58294532	A	intragenic	3.24E-01	**1.83E-06**	6.96E-06
rs6986207	8:58294520	C	intragenic	3.09E-01	**1.83E-06**	6.44E-06
rs72652957	8:58292501	T	intragenic	2.90E-01	**1.83E-06**	5.51E-06
rs10071534	5:42922073	C	intragenic	2.79E-01	**1.95E-06**	8.02E-04
rs10063717	5:42922110	C	intragenic	2.70E-01	**2.01E-06**	9.08E-04
rs3901254	6:37659300	A	*MDGA1*	6.22E-01	**2.05E-06**	4.64E-05
rs72652956	8:58291736	C	intragenic	2.56E-01	**2.14E-06**	4.82E-06
rs16922102	8:58302927	T	intragenic	5.09E-01	**2.23E-06**	2.21E-05
rs16922104	8:58302952	G	intragenic	5.09E-01	**2.23E-06**	2.21E-05
rs17031805	3:34546401	G	intragenic	1.38E-01	**2.26E-06**	3.36E-06
rs57744550	8:58294704	G	intragenic	3.24E-01	**2.28E-06**	8.28E-06
rs16922105	8:58303150	T	intragenic	4.73E-01	**2.32E-06**	1.95E-05
rs56924116	8:58303266	A	intragenic	4.85E-01	**2.32E-06**	2.05E-05

rs7899845	10:21265593	C	*NEBL*	8.08E-01	**2.37E-06**	8.98E-05
rs7814333	8:58302524	G	intragenic	5.09E-01	**2.44E-06**	2.36E-05
rs7826185	8:58301947	G	intragenic	5.09E-01	**2.44E-06**	2.36E-05
rs56832078	8:58293711	A	intragenic	4.09E-01	**2.53E-06**	1.40E-05
rs16922088	8:58298072	T	intragenic	5.66E-01	**2.59E-06**	3.06E-05
rs11990740	8:58297248	A	intragenic	5.38E-01	**2.63E-06**	2.78E-05
rs57945649	8:58298471	C	intragenic	5.45E-01	**2.63E-06**	2.87E-05
rs10756292	9:11814070	G	intragenic	9.47E-01	**2.81E-06**	2.63E-04
rs9346746	6:158726711	C	*TULP4*	8.06E-02	**2.91E-06**	3.07E-06
rs6919284	6:158726917	C	*TULP4*	7.83E-02	**2.96E-06**	2.97E-06
rs11994423	8:58296966	G	intragenic	5.32E-01	**3.10E-06**	3.09E-05
rs11994454	8:58297107	C	intragenic	5.35E-01	**3.10E-06**	3.12E-05
rs11994487	8:58297238	A	intragenic	5.38E-01	**3.10E-06**	3.15E-05
rs11997856	8:58297281	G	intragenic	5.39E-01	**3.10E-06**	3.16E-05
rs16922086	8:58298006	G	intragenic	5.52E-01	**3.10E-06**	3.33E-05
rs16922090	8:58301045	G	intragenic	5.08E-01	**3.10E-06**	2.80E-05
rs4738588	8:58296951	A	intragenic	5.32E-01	**3.10E-06**	3.08E-05
rs4738590	8:58300087	T	intragenic	5.20E-01	**3.10E-06**	2.92E-05
rs4738591	8:58300482	G	intragenic	5.20E-01	**3.10E-06**	2.92E-05
rs4738593	8:58300580	G	intragenic	5.19E-01	**3.10E-06**	2.91E-05
rs55861231	8:58295839	T	intragenic	5.11E-01	**3.10E-06**	2.85E-05
rs56374955	8:58295747	C	intragenic	5.10E-01	**3.10E-06**	2.84E-05
rs57829000	8:58301000	T	intragenic	5.08E-01	**3.10E-06**	2.80E-05
rs58077807	8:58298095	G	intragenic	5.54E-01	**3.10E-06**	3.36E-05
rs58748161	8:58301217	A	intragenic	5.09E-01	**3.10E-06**	2.80E-05
rs59643894	8:58300172	T	intragenic	5.20E-01	**3.10E-06**	2.92E-05
rs60129174	8:58297827	T	intragenic	5.49E-01	**3.10E-06**	3.29E-05
rs60999477	8:58301231	G	intragenic	5.09E-01	**3.10E-06**	2.80E-05
rs72652967	8:58296134	G	intragenic	5.17E-01	**3.10E-06**	2.91E-05
rs72652974	8:58299721	G	intragenic	5.20E-01	**3.10E-06**	2.93E-05
rs72652975	8:58299980	G	intragenic	5.20E-01	**3.10E-06**	2.92E-05
rs9694509	8:58301660	C	intragenic	5.09E-01	**3.10E-06**	2.80E-05
rs9694545	8:58301685	A	intragenic	5.09E-01	**3.10E-06**	2.80E-05

rs7915328	10:21281165	C	*NEBL*	5.42E-01	**3.37E-06**	1.89E-04
rs72652966	8:58295621	G	intragenic	5.84E-01	**3.39E-06**	4.13E-05
rs9346748	6:158727843	G	*TULP4*	7.64E-02	**3.53E-06**	3.30E-06
rs72652983	8:58303405	C	intragenic	5.42E-01	**3.62E-06**	3.73E-05
rs2358597	3:34655837	G	intragenic	8.13E-01	**3.77E-06**	3.48E-04
rs72652964	8:58295466	C	intragenic	5.54E-01	**4.04E-06**	4.02E-05
rs72652965	8:58295490	G	intragenic	5.54E-01	**4.04E-06**	4.02E-05
rs1891353	6:158733024	C	*TULP4*	1.01E-01	**4.13E-06**	5.63E-06
rs10080516	6:158719296	A	*TULP4*	1.17E-01	**4.15E-06**	6.38E-06
rs11012445	10:21268612	G	*NEBL*	4.89E-01	**4.18E-06**	1.19E-03
rs4738589	8:58300037	G	intragenic	5.82E-01	**4.23E-06**	4.63E-05
rs13177180	5:114880593	A	*FEM1C*	5.61E-01	**4.52E-06**	5.95E-05
rs11758177	6:158731038	A	*TULP4*	7.98E-02	**4.59E-06**	4.35E-06
rs6455575	6:158747305	A	*TULP4*	7.77E-02	**4.92E-06**	4.33E-06
rs11994338	8:58298729	G	intragenic	6.12E-01	**5.08E-06**	5.83E-05
rs72652982	8:58303173	G	intragenic	6.12E-01	**5.09E-06**	6.06E-05
rs62439867	6:158710801	T	*TULP4*	1.01E-01	**5.14E-06**	6.02E-06
rs2096164	6:158731340	G	*TULP4*	7.97E-02	**5.31E-06**	4.87E-06
rs55837297	8:58295907	C	intragenic	5.12E-01	**5.41E-06**	4.38E-05
rs73756772	5:56002136	C	intragenic	5.60E-01	**5.43E-06**	1.53E-03
rs60375467	10:21268789	T	*NEBL*	4.84E-01	**5.66E-06**	1.43E-03
rs67955617	10:21268678	C	*NEBL*	4.86E-01	**5.66E-06**	1.42E-03
rs7914197	10:21269587	C	*NEBL*	4.75E-01	**5.66E-06**	1.48E-03
rs7917492	10:21269512	C	*NEBL*	4.75E-01	**5.66E-06**	1.48E-03
rs827863	6:158710913	C	*TULP4*	1.01E-01	**5.80E-06**	6.61E-06
rs827866	6:158714409	T	*TULP4*	1.01E-01	**5.80E-06**	6.61E-06
rs827867	6:158714489	G	*TULP4*	1.01E-01	**5.80E-06**	6.61E-06
rs827868	6:158714879	C	*TULP4*	1.01E-01	**5.80E-06**	6.61E-06
rs57334606	8:58295900	G	intragenic	5.12E-01	**6.57E-06**	5.08E-05
rs58863401	8:58295898	C	intragenic	5.12E-01	**6.57E-06**	5.08E-05
rs67108474	3:34586257	G	*LOC102724048*	2.58E-01	**6.58E-06**	1.63E-05
rs55771104	8:58298853	A	intragenic	6.11E-01	**6.83E-06**	7.30E-05
rs169716	6:11961951	A	intragenic	3.80E-01	**6.88E-06**	3.04E-05

rs11816696	10:25362890	C	intragenic	9.48E-01	**6.90E-06**	5.14E-04
rs9364933	6:158722326	T	*TULP4*	9.49E-02	**6.92E-06**	7.19E-06
rs13390546	2:29946273	A	*ALK*	8.67E-01	**6.99E-06**	2.71E-04
rs56314493	8:58298854	C	intragenic	6.11E-01	**7.01E-06**	7.44E-05
rs4709185	6:158728496	T	*TULP4*	8.46E-02	**7.13E-06**	6.61E-06
rs1489696	2:18533458	A	intragenic	7.71E-01	**7.14E-06**	9.95E-04
rs827869	6:158714993	G	*TULP4*	1.01E-01	**7.15E-06**	7.77E-06
rs827870	6:158715050	G	*TULP4*	1.01E-01	**7.15E-06**	7.77E-06
rs827871	6:158715312	G	*TULP4*	1.01E-01	**7.15E-06**	7.77E-06
rs827872	6:158715755	A	*TULP4*	1.01E-01	**7.15E-06**	7.77E-06
rs827873	6:158716281	G	*TULP4*	1.03E-01	**7.15E-06**	7.88E-06
rs7728625	5:43058296	G	*LOC648987*	3.37E-01	**7.47E-06**	1.09E-03
rs3847797	12:94544545	T	*PLXNC1*	5.69E-01	**7.63E-06**	1.96E-04
rs827865	6:158714124	G	*TULP4*	1.31E-01	**7.66E-06**	1.21E-05
rs7541623	1:204859749	G	*NFASC*	1.07E-01	**7.69E-06**	8.48E-06
rs9456275	6:158719620	C	*TULP4*	8.71E-02	**7.75E-06**	6.70E-06
rs862411	6:158710230	G	*TULP4*	1.01E-01	**7.77E-06**	8.28E-06
rs13074290	3:34548878	G	intragenic	2.71E-01	**7.78E-06**	7.88E-06
rs57373979	6:158708899	C	*TULP4*	1.01E-01	**7.97E-06**	8.43E-06
rs57756309	6:158708940	A	*TULP4*	1.01E-01	**7.97E-06**	8.43E-06
rs827859	6:158708455	G	*TULP4*	1.01E-01	**7.97E-06**	8.43E-06
rs4709187	6:158728946	G	*TULP4*	7.42E-02	**8.03E-06**	6.11E-06
rs10828163	10:21266827	C	*NEBL*	5.05E-01	**8.07E-06**	1.56E-03
rs4709186	6:158728764	T	*TULP4*	7.45E-02	**8.29E-06**	6.28E-06
rs11012446	10:21269710	G	*NEBL*	4.75E-01	**8.45E-06**	1.90E-03
rs11012447	10:21269977	C	*NEBL*	4.80E-01	**8.45E-06**	1.87E-03
rs9364934	6:158722389	A	*TULP4*	1.26E-01	**8.58E-06**	1.25E-05
rs10050856	5:23371640	T	intragenic	1.92E-01	**8.82E-06**	1.23E-05
rs827853	6:158705473	C	*TULP4*	1.01E-01	**8.82E-06**	9.03E-06
rs827854	6:158705663	G	*TULP4*	1.01E-01	**8.82E-06**	9.04E-06
rs56121699	8:58294750	A	intragenic	3.51E-01	**8.86E-06**	2.88E-05
rs36040276	17:11211810	A	*SHISA6*	4.58E-01	**9.09E-06**	1.35E-03
rs35946663	14:96829998	A	intragenic	2.53E-01	**9.19E-06**	2.76E-05

rs2153808	6:158757258	T	*TULP4*	6.15E-02	**9.34E-06**	5.26E-06
rs960941	10:21271153	G	*NEBL*	4.94E-01	**9.45E-06**	1.79E-03
rs60280851	15:68959002	A	*CORO2B*	3.98E-02	**9.75E-06**	1.93E-06
rs12629506	3:34556106	T	intragenic	1.92E-01	**9.89E-06**	1.78E-05
**All cohorts combined**						
rs13102150	4:143470133	C	*INPP4B*	1.83E-04	7.37E-04	**6.43E-07**
rs921541	15:74160103	C	*LOC102723657*	2.10E-01	1.65E-07	**9.28E-07**
rs3843430	4:14101273	T	intragenic	3.33E-05	3.67E-03	**1.16E-06**
rs11100748	4:143460923	A	*INPP4B*	5.00E-04	9.02E-04	**1.75E-06**
rs60280851	15:68959002	G	*CORO2B*	3.98E-02	9.75E-06	**1.93E-06**
rs1923665	1:204864152	A	*NFASC*	1.22E-02	4.44E-05	**2.00E-06**
rs12504375	4:143456800	C	*INPP4B*	6.86E-04	9.20E-04	**2.34E-06**
rs34054994	15:68958238	G	*CORO2B*	4.02E-02	1.40E-05	**2.66E-06**
rs4975317	4:143457845	T	*INPP4B*	8.03E-04	9.42E-04	**2.76E-06**
rs6835296	4:143455287	A	*INPP4B*	1.33E-02	5.81E-05	**2.76E-06**
rs911988	1:204863306	T	*NFASC*	1.24E-02	6.44E-05	**2.85E-06**
rs2351027	4:112790034	C	*LOC105377369*	6.21E-03	1.44E-04	**2.89E-06**
rs13353396	3:34547165	T	intragenic	3.16E-01	2.61E-07	**2.95E-06**
rs6919284	6:158726917	C	*TULP4*	7.83E-02	2.96E-06	**2.97E-06**
rs9346746	6:158726711	C	*TULP4*	8.06E-02	2.91E-06	**3.07E-06**
rs11930912	4:143461480	T	*INPP4B*	5.73E-04	1.38E-03	**3.18E-06**
rs9346748	6:158727843	G	*TULP4*	7.64E-02	3.53E-06	**3.30E-06**
rs17031805	3:34546401	A	intragenic	1.38E-01	2.26E-06	**3.36E-06**
rs17016344	4:143469061	G	*INPP4B*	2.12E-04	3.34E-03	**3.55E-06**
rs58899027	4:143465318	A	*INPP4B*	1.59E-04	4.33E-03	**3.81E-06**
rs1852808	3:34556501	C	intragenic	2.45E-01	7.04E-07	**3.89E-06**
rs4246729	4:143468371	G	*INPP4B*	1.99E-04	3.85E-03	**3.99E-06**
rs17278757	15:68960258	C	*CORO2B*	5.80E-02	1.55E-05	**4.24E-06**
rs4101198	4:14044759	G	intragenic	4.77E-04	1.89E-03	**4.24E-06**
rs6455575	6:158747305	A	*TULP4*	7.77E-02	4.92E-06	**4.33E-06**
rs11758177	6:158731038	A	*TULP4*	7.98E-02	4.59E-06	**4.35E-06**
rs6847349	4:14040923	G	intragenic	1.39E-04	4.36E-03	**4.38E-06**
rs1425518	4:143462437	C	*INPP4B*	1.04E-04	6.71E-03	**4.56E-06**

rs1476122	4:143469236	G	*INPP4B*	2.12E-04	4.26E-03	**4.71E-06**
rs72652956	8:58291736	A	intragenic	2.56E-01	2.14E-06	**4.82E-06**
rs2096164	6:158731340	G	*TULP4*	7.97E-02	5.31E-06	**4.87E-06**
rs3801321	7:27279191	T	intragenic	2.67E-04	3.39E-03	**5.02E-06**
rs6996802	8:58293378	T	intragenic	3.00E-01	1.49E-06	**5.13E-06**
rs7018443	8:58293505	A	intragenic	3.02E-01	1.49E-06	**5.21E-06**
rs2153808	6:158757258	T	*TULP4*	6.15E-02	9.34E-06	**5.26E-06**
rs4975306	4:143453881	A	*INPP4B*	1.37E-03	1.12E-03	**5.27E-06**
rs2059511	4:143460080	A	*INPP4B*	5.78E-04	2.30E-03	**5.41E-06**
rs1535478	9:7990651	T	intragenic	2.94E-05	1.55E-02	**5.43E-06**
rs9456284	6:158732397	G	*TULP4*	5.12E-02	1.41E-05	**5.46E-06**
rs4975313	4:143468733	G	*INPP4B*	2.06E-04	4.96E-03	**5.49E-06**
rs79391134	4:143460250	T	*INPP4B*	6.46E-04	2.13E-03	**5.50E-06**
rs72652957	8:58292501	C	intragenic	2.90E-01	1.83E-06	**5.51E-06**
rs6816875	4:143464033	T	*INPP4B*	1.33E-04	6.70E-03	**5.56E-06**
rs1891353	6:158733024	C	*TULP4*	1.01E-01	4.13E-06	**5.63E-06**
rs11100749	4:143461463	G	*INPP4B*	6.35E-04	2.26E-03	**5.78E-06**
rs2059510	4:143459907	C	*INPP4B*	6.34E-04	2.30E-03	**5.84E-06**
rs2217016	4:143464619	G	*INPP4B*	1.40E-04	6.81E-03	**5.91E-06**
rs6996833	8:58293434	T	intragenic	3.00E-01	1.77E-06	**5.96E-06**
rs2059513	4:143460462	G	*INPP4B*	5.62E-04	2.59E-03	**6.01E-06**
rs62439867	6:158710801	C	*TULP4*	1.01E-01	5.14E-06	**6.02E-06**
rs4709187	6:158728946	G	*TULP4*	7.42E-02	8.03E-06	**6.11E-06**
rs7671198	4:143470929	T	*INPP4B*	2.58E-04	4.69E-03	**6.17E-06**
rs16998558	4:143471217	T	*INPP4B*	2.68E-04	4.65E-03	**6.28E-06**
rs4709186	6:158728764	T	*TULP4*	7.45E-02	8.29E-06	**6.28E-06**
rs10080516	6:158719296	A	*TULP4*	1.17E-01	4.15E-06	**6.38E-06**
rs6981267	8:58293977	A	intragenic	3.09E-01	1.83E-06	**6.41E-06**
rs6981437	8:58294054	A	intragenic	3.09E-01	1.83E-06	**6.41E-06**
rs6981568	8:58293936	G	intragenic	3.09E-01	1.83E-06	**6.41E-06**
rs55960888	8:58294130	A	intragenic	3.09E-01	1.83E-06	**6.42E-06**
rs17016354	4:143472696	T	*INPP4B*	2.68E-04	4.75E-03	**6.43E-06**
rs6986207	8:58294520	G	intragenic	3.09E-01	1.83E-06	**6.44E-06**

rs17717651	4:143453079	A	*INPP4B*	1.35E-03	1.37E-03	**6.45E-06**
rs4975318	4:143454105	C	*INPP4B*	1.20E-03	1.54E-03	**6.54E-06**
rs4709185	6:158728496	T	*TULP4*	8.46E-02	7.13E-06	**6.61E-06**
rs827863	6:158710913	T	*TULP4*	1.01E-01	5.80E-06	**6.61E-06**
rs827866	6:158714409	C	*TULP4*	1.01E-01	5.80E-06	**6.61E-06**
rs827867	6:158714489	A	*TULP4*	1.01E-01	5.80E-06	**6.61E-06**
rs827868	6:158714879	T	*TULP4*	1.01E-01	5.80E-06	**6.61E-06**
rs12504770	4:143452719	T	*INPP4B*	1.36E-03	1.40E-03	**6.64E-06**
rs9456275	6:158719620	C	*TULP4*	8.71E-02	7.75E-06	**6.70E-06**
rs6985947	8:58294532	C	intergenic	3.24E-01	1.83E-06	**6.96E-06**
rs10900429	1:204860968	G	*NFASC*	1.03E-02	2.16E-04	**7.06E-06**
rs7676337	4:14038869	G	intergenic	2.59E-04	4.41E-03	**7.13E-06**
rs9364933	6:158722326	T	*TULP4*	9.49E-02	6.92E-06	**7.19E-06**
rs6537109	4:143473479	A	*INPP4B*	4.02E-04	3.88E-03	**7.33E-06**
rs12405099	1:204861083	C	*NFASC*	1.02E-02	2.27E-04	**7.37E-06**
rs1891352	6:158733562	C	*TULP4*	7.83E-02	1.04E-05	**7.39E-06**
rs7741924	6:158733310	A	*TULP4*	7.83E-02	1.04E-05	**7.39E-06**
rs3846368	4:14056459	G	intergenic	3.54E-04	3.80E-03	**7.47E-06**
rs1373381	4:14057145	T	intergenic	3.54E-04	3.80E-03	**7.48E-06**
rs3857159	4:14056930	T	intergenic	3.54E-04	3.80E-03	**7.48E-06**
rs4975316	4:143457985	A	*INPP4B*	9.24E-04	2.18E-03	**7.60E-06**
rs9347228	6:158735905	C	*TULP4*	6.44E-02	1.51E-05	**7.61E-06**
rs3846367	4:14056393	T	intergenic	3.32E-04	4.03E-03	**7.65E-06**
rs7756620	6:158735090	T	*TULP4*	7.11E-02	1.30E-05	**7.68E-06**
rs9364951	6:158735087	C	*TULP4*	7.11E-02	1.30E-05	**7.68E-06**
rs827869	6:158714993	A	*TULP4*	1.01E-01	7.15E-06	**7.77E-06**
rs827870	6:158715050	A	*TULP4*	1.01E-01	7.15E-06	**7.77E-06**
rs827871	6:158715312	A	*TULP4*	1.01E-01	7.15E-06	**7.77E-06**
rs827872	6:158715755	T	*TULP4*	1.01E-01	7.15E-06	**7.77E-06**
rs13074290	3:34548878	T	intragenic	2.71E-01	7.78E-06	**7.88E-06**
rs827873	6:158716281	A	*TULP4*	1.03E-01	7.15E-06	**7.88E-06**
rs9346743	6:158722673	A	*TULP4*	7.69E-02	1.23E-05	**7.88E-06**
rs9457351	6:158760899	A	*TULP4*	7.71E-02	1.19E-05	**8.00E-06**

rs9355646	6:158726194	G	*TULP4*	8.18E-02	1.09E-05	**8.05E-06**
rs6982211	8:58294313	G	intragenic	3.51E-01	1.82E-06	**8.14E-06**
rs57942103	8:106513461	A	*ZFPM2*	6.91E-02	1.69E-05	**8.17E-06**
rs13221828	7:27271127	C	intragenic	2.95E-04	4.98E-03	**8.27E-06**
rs57744550	8:58294704	A	intragenic	3.24E-01	2.28E-06	**8.28E-06**
rs862411	6:158710230	A	*TULP4*	1.01E-01	7.77E-06	**8.28E-06**
rs6455564	6:158729023	C	*TULP4*	7.37E-02	1.25E-05	**8.39E-06**
rs57373979	6:158708899	T	*TULP4*	1.01E-01	7.97E-06	**8.43E-06**
rs57756309	6:158708940	T	*TULP4*	1.01E-01	7.97E-06	**8.43E-06**
rs827859	6:158708455	T	*TULP4*	1.01E-01	7.97E-06	**8.43E-06**
rs7541623	1:204859749	A	*NFASC*	1.07E-01	7.69E-06	**8.48E-06**
rs61104737	4:143455438	C	*INPP4B*	9.15E-04	2.45E-03	**8.54E-06**
rs12504378	4:143456826	C	*INPP4B*	8.01E-04	2.74E-03	**8.63E-06**
rs60951295	4:143475473	C	*INPP4B*	1.52E-04	7.95E-03	**8.75E-06**
rs10519649	4:143454553	C	*INPP4B*	1.14E-03	2.13E-03	**8.78E-06**
rs3906860	4:14054875	T	intragenic	3.87E-04	4.17E-03	**8.92E-06**
rs4433000	6:158734275	C	*TULP4*	7.03E-02	1.60E-05	**8.92E-06**
rs12405378	1:204861371	C	*NFASC*	1.02E-02	2.82E-04	**9.02E-06**
rs827853	6:158705473	T	*TULP4*	1.01E-01	8.82E-06	**9.03E-06**
rs827854	6:158705663	A	*TULP4*	1.01E-01	8.82E-06	**9.04E-06**
rs4708788	6:158760208	T	*TULP4*	5.90E-02	2.25E-05	**9.13E-06**
rs7122295	11:59050845	G	intragenic	1.22E-02	2.50E-04	**9.14E-06**
rs9456293	6:158760776	A	*TULP4*	6.64E-02	1.84E-05	**9.19E-06**
rs11244263	9:133931123	C	*LAMC3*	4.65E-02	4.83E-03	**9.21E-06**
rs6827084	4:14058506	T	intragenic	3.64E-04	4.50E-03	**9.28E-06**
rs7746246	6:158725549	C	*TULP4*	8.33E-02	1.35E-05	**9.49E-06**
rs4484375	4:143450615	C	*INPP4B*	1.01E-02	3.05E-04	**9.52E-06**
rs12405381	1:204861384	C	*NFASC*	1.02E-02	3.02E-04	**9.60E-06**
rs3944007	16:49004284	G	intragenic	5.62E-03	5.57E-04	**9.69E-06**
rs4785155	16:49003778	C	intragenic	5.15E-03	6.08E-04	**9.72E-06**
rs3812100	6:158732534	T	*TULP4*	7.65E-02	1.47E-05	**9.80E-06**
rs79379713	11:60716249	A	*SLC15A3*	9.62E-01	3.30E-04	**9.82E-06**
rs828010	6:158772389	T	*TULP4*	7.71E-02	1.56E-05	**9.89E-06**

rs4994220	6:158753282	T	*TULP4*	6.93E-02	1.88E-05	**9.91E-06**
rs6941081	6:158756681	A	*TULP4*	6.97E-02	1.88E-05	**9.99E-06**

Supplemental Table S3: Genes with suggestive evidence of association (p<1.0 x 10^{-3})

Gene name	Chr†	Start	Stop	Cohort with 22q.11.2 deletion syndrome	Cohort without 22.q.11.2 deletion syndrome	All cohorts combined
GLYATL3	6	49466671	49496777	**5.56E-05**	2.60E-01	1.20E-03
GSDMD	8	144634383	144646232	**6.67E-05**	8.00E-01	8.30E-02
NGB	14	77730834	77738655	**9.17E-05**	8.91E-01	3.70E-02
C4orf36	4	87796358	87825820	**1.83E-04**	6.94E-01	2.30E-02
HIST1H1C	6	26054968	26057699	**3.33E-04**	4.01E-01	5.98E-01
TINAG	6	54172203	54255950	**5.00E-04**	6.34E-01	3.20E-02
ZC3H3	8	144518825	144625449	**5.00E-04**	6.53E-01	1.08E-01
FCER2	19	7752643	7768036	**5.67E-04**	7.62E-01	8.60E-02
METTL13	1	171749761	171767856	**6.50E-04**	6.68E-01	7.60E-03
HIST1H2BC	6	26112389	26125266	**6.67E-04**	9.45E-01	2.03E-01
SPOCK3	4	167653535	168156741	**7.50E-04**	2.02E-01	1.86E-02
MYPN	10	69864874	69972774	**7.50E-04**	6.49E-01	3.39E-01
LAMC3	9	133883504	133969446	**9.50E-04**	7.50E-02	**1.83E-04**
ITGB3BP	1	63905441	63989944	3.22E-01	**3.67E-04**	1.70E-02
ZC3H11A	1	203763665	203824256	9.04E-01	**5.00E-04**	1.40E-02
ZBED6	1	203765651	203770590	7.89E-01	**2.50E-04**	1.10E-02
FEM1C	5	114855605	114881591	9.07E-01	**5.22E-05**	**5.50E-04**
TICAM2	5	114913339	114953142	7.54E-01	**3.67E-04**	**6.50E-04**
TMED7-TICAM2	5	114913339	114962876	7.51E-01	**1.83E-04**	**5.00E-04**
TMED7	5	114947905	114962876	7.16E-01	**2.50E-04**	2.00E-03
TFAP2A	6	10392419	10420797	5.61E-01	**7.50E-04**	3.50E-02
TULP4	6	158652268	158933860	3.20E-01	**3.33E-04**	**3.33E-04**
PIWIL2	8	22131810	22216560	1.80E-01	**6.00E-04**	**1.43E-04**
AGTPBP1	9	88160454	88357944	1.80E-01	**4.00E-04**	**1.43E-04**

ADARB2	10	1222253	1780670	5.84E-01	**6.50E-04**	1.10E-01
CEP55	10	95255369	95289849	2.06E-01	**3.50E-04**	1.40E-03
OR51B2	11	5343528	5346582	5.62E-01	**7.00E-04**	6.20E-03
ZBED5	11	10873251	10880620	3.23E-01	**6.00E-04**	3.40E-03
TMPRSS13	11	117770356	117801168	5.51E-01	**7.50E-04**	1.50E-02
KHNYN	14	24897882	24911548	2.09E-01	**1.22E-04**	3.80E-02
SDR39U1	14	24907972	24913042	1.42E-01	**2.20E-04**	6.40E-02
CALM1	14	90862327	90875619	9.93E-01	**7.00E-04**	6.80E-03
ATG2B	14	96746595	96830678	1.30E-01	**6.00E-04**	**7.50E-04**
GSKIP	14	96828789	96854627	7.35E-01	**2.75E-04**	1.40E-03
CORO2B	15	68850614	69021145	4.97E-01	**3.50E-04**	**2.00E-04**
TBC1D21	15	74164922	74182555	1.43E-01	**3.23E-05**	1.60E-02
ATF7IP2	16	10478912	10578495	2.34E-01	**2.50E-04**	**1.43E-04**
SHISA6	17	11143740	11468380	2.25E-01	**6.47E-05**	1.48E-02
IGL	22	22379474	23266085	3.82E-01	**9.50E-04**	**7.50E-04**
CLIC4	1	25070760	25171815	1.60E-02	6.60E-02	**9.50E-04**
CASQ2	1	116241624	116312426	1.90E-02	4.70E-02	**6.50E-04**
NFASC	1	204796782	204992950	4.07E-01	1.50E-03	**3.25E-04**
INPP4B	4	142948181	143768604	1.60E-02	4.40E-02	**5.00E-04**
ARHGAP26	5	142148881	142609572	4.60E-02	1.70E-02	**6.00E-04**
EVX1	7	27281164	27288438	2.40E-03	4.20E-02	**8.33E-05**
SLC35G5	8	11187495	11190695	4.30E-02	4.60E-03	**5.00E-04**
DEFB134	8	11850489	11854760	1.10E-02	4.70E-02	**6.00E-04**
PTCH1	9	98204264	98280247	3.10E-02	1.10E-02	**7.00E-04**
CLRN3	10	129675114	129692211	7.71E-01	4.20E-03	**5.50E-04**
OR5T2	11	55998582	56001661	2.15E-01	4.20E-03	**8.00E-04**
TAGLN	11	117069040	117076508	8.40E-02	6.40E-03	**7.50E-04**

UPK2	11	118826008	118830269	1.00E-02	5.90E-02	**6.00E-04**
STAB2	12	103980069	104161502	2.50E-02	1.12E-02	**8.50E-04**

† Chromosome

Supplemental Table S4: Summary data for genes located in the 22q.11.2 deletion region (chr22:14,700,001-25,900,000) among the cohort without 22q11.2DS, sorted by p-value.

Gene Name (Gene Symbol)	Chr†	Start‡	Stop	p-value
Histone Cell Cycle Regulator (HIRA)	22	19318221	19419247	2.70E-02
Testis Specific Serine Kinase 2 (TSSK2)	22	19118321	19120136	3.10E-02
DiGeorge Syndrome Critical Region Gene 6 (DGCR6)	22	18893736	18899601	3.20E-02
Proline Dehydrogenase 1 (PRODH)	22	18900206	18924066	4.40E-02
Zinc Finger DHHC-Type Palmitoyltransferase 8 (ZDHHC8)	22	20119364	20135530	5.00E-02
CRK Like Proto-Oncogene, Adaptor Protein (CRKL)	22	21271714	21308037	6.40E-02
Reticulon 4 Receptor (RTN4R)	22	20228938	20255816	6.40E-02
Ess-2 Splicing Factor Homolog (DGCR14)	22	19117792	19132190	8.30E-02
DiGeorge Syndrome Critical Region Gene 2 (DGCR2)	22	19023795	19109967	1.00E-01
DGCR8 Microprocessor Complex Subunit (DGCR8)	22	20067755	20099400	1.03E-01
Serpin Family D Member 1 (SERPIND1)	22	21128383	21142008	1.35E-01
G Protein Subunit Beta 1 Like (GNB1L)	22	19775932	19842462	1.37E-01
Leucine Zipper Like Transcription Regulator 1 (LZTR1)	22	21336558	21353326	1.60E-01
Transport and Golgi Organization 2 Homolog (TANGO2)	22	20004554	20053449	1.66E-01
Mitochondrial Ribosomal Protein L40 (MRPL40)	22	19419302	19423600	1.93E-01
RAN Binding Protein 1 (RANBP1)	22	20103461	20114880	1.97E-01
TRNA Methyltransferase 2 Homolog A (TRMT2A)	22	20099389	20104818	2.08E-01
Claudin 5 (CLDN5)	22	19510547	19515068	2.11E-01
Phosphatidylinositol 4-Kinase Alpha (PI4KA)	22	21061979	21213100	2.19E-01
Synaptosome Associated Protein 29 (SNAP29)	22	21213292	21245502	2.39E-01
Chromosome 22 Open Reading Frame 39 (C22orf39)	22	19428409	19435755	3.33E-01

Retrotransposon Gag Like 10 (C22orf29)	22	19833661	19842371	3.49E-01
Cell Division Cycle 45 (CDC45)	22	19466984	19508135	4.14E-01
T-Box Transcription Factor 1 (TBX1)	22	19744226	19771116	4.15E-01
Thioredoxin Reductase 2 (TXNRD2)	22	19863040	19929359	4.15E-01
DiGeorge Syndrome Critical Region Gene 6 Like (DGCR6L)	22	20301761	20307628	4.19E-01
Zinc Finger Protein 74 (ZNF74)	22	20748405	20762753	4.96E-01
THAP Domain Containing 7 (THAP7)	22	21354061	21356404	5.31E-01
Apoptosis Inducing Factor Mitochondria Associated 3 (AIFM3)	22	21319418	21335649	5.67E-01
Leucine Rich Repeat Containing 74B (LRRC74B)	22	21400249	21418457	5.98E-01
HIC ZBTB Transcriptional Repressor 2 (HIC2)	22	21771693	21805752	5.98E-01
Ubiquitin Recognition Factor in ER Associated Degradation 1 (UFD1L)	22	19437464	19466818	6.29E-01
Purinergic Receptor P2X 6 (P2RX6)	22	21369442	21382302	7.16E-01
Goosecoid Homeobox 2 (GSC2)	22	19136504	19137796	7.93E-01
ARVCF Delta Catenin Family Member (ARVCF)	22	19954126	20004325	8.07E-01
Mediator Complex Subunit 15 (MED15)	22	20861849	20941919	8.08E-01
Kelch Like Family Member 22 (KLHL22)	22	20795806	20850170	8.43E-01
Solute Carrier Family 25 Member 1 (SLC25A1)	22	19163088	19166338	8.93E-01
Clathrin Heavy Chain Like 1 (CLTCL1)	22	19166986	19279247	9.46E-01
Catechol-O-Methyltransferase (COMT)	22	19929263	19957498	9.55E-01
Solute Carrier Family 7 Member 4 (SLC7A4)	22	21382995	21387163	9.65E-01
Scavenger Receptor Class F Member 2 (SCARF2)	22	20778874	20792146	9.86E-01

† Chromosome

‡ Genome Reference Consortium Human genome build 37/hg19 reference assembly
